# Coq10 for preventing cardiotoxicity in breast cancer patients treated with trastuzumab

**DOI:** 10.25122/jml-2023-0098

**Published:** 2023-08

**Authors:** Nawal Al-Hammadi, Emad AlSabri, Ahmed Hassan Kudhair, Heider Qassam, Najah Rayish Hadi

**Affiliations:** 1 Kufa Technical Institute, Al-Furat Al-Awsat Technical University, Najaf, Iraq; 2 Middle Euphrates Cancer Therapy Center, Najaf, Iraq; 3 Department of Biochemistry, Faculty of Medicine, Jabir Ibn Hayyan Medical University, Najaf, Iraq; 4 Department of Pharmacology and Therapeutics, Faculty of Medicine, University of Kufa, Iraq

**Keywords:** Trastuzumab, Cyclophosphamide, Docetaxel, trastuzumab loading dosage

## Abstract

Trastuzumab is a successful treatment option for HER2-positive breast cancer, but a decline in left ventricular ejection fraction (LVEF) and an increase in inflammatory and cardiac enzyme biomarkers can lead to cessation and termination of therapy. This study aimed to investigate the ability of Coenzyme Q10 (Coq10) to avoid these adverse effects. The study included 100 female patients with HER2+ (HER2+3 or amplified gene) breast cancer. All patients underwent standard adjuvant chemotherapy regimens, which involved a four-cycle treatment of Adriamycin, Cyclophosphamide, Docetaxel, and an initial 8 mg/kg loading dose of trastuzumab, followed by a year of 6 mg/kg maintenance doses every three weeks. One group of 50 patients received trastuzumab and a placebo, while the other 50 were given trastuzumab and CoQ10 for a full year. The CoQ10-treated group exhibited a statistically significant decrease in levels of monocyte chemoattractant protein-1 (MCP-1), interleukin-6 (IL6), soluble toll-like receptor 4 (sTLR4), and cardiac troponin I (cTnI) compared to the control group (p<0.05). However, there was no significant difference in the mean F2-isoprostane levels between the treated and the control groups at any data collection point. Furthermore, the CoQ10-treated group experienced a significant reduction in the decline of EF levels compared to the control group at all stages except for baseline. According to our findings, Coenzyme Q10 protected patients with HER2+3 breast cancer from the cardiotoxicity of trastuzumab by increasing ejection fraction and decreasing inflammatory biomarkers and cardiac enzyme levels.

## INTRODUCTION

Breast cancer represents a significant global concern, with an alarming 2.1 million new cases diagnosed annually. It predominantly affects women, accounting for 24.2% of cancer diagnoses in this demographic. Breast cancer affects one in four women worldwide, contributing to 15% of fatalities [1]. The current gold standard of care for patients with HER2-positive (HER2+) breast cancer is trastuzumab in combination with pertuzumab, a humanized monoclonal antibody leading to inhibition of the dimerization of HER2 [2, 3]. While trastuzumab considerably increases the chances of survival, several patients have been compelled to stop treatment because they later acquired heart failure.

The mechanism behind trastuzumab-induced cardiotoxicity is linked to alterations in the antiapoptotic signaling pathways in cardiomyocytes that cause congestive heart failure during in vivo studies of acute chemotherapy-induced cardiac dysfunction [4, 5]. This is further mediated through the modification of the NADPH oxidase and mitogen-activated protein kinase (MAPK) signaling pathways. Other investigations have connected the renin-angiotensin system, especially angiotensin II (ANG II) signaling, to trastuzumab-induced cardiac failure [6, 7]. Moreover, modification of HER2 signaling via MAPKs and NADPH oxidase results in heightened oxidative stress and dilated cardiomyopathy [6]. Trastuzumab-induced cardiotoxicity may manifest as symptomatic heart failure or asymptomatic decline in left ventricular ejection fraction (LVEF) [8], with no apparent correlation to dosage or timing [9]. As a result, early discontinuation of the drug can reverse its cardiac effects, often with the assistance of medication [10].

Coenzyme Q10 (CoQ10), also known as ubiquinone, is a vitamin-like benzoquinone molecule naturally synthesized within the human body that is essential for energy production in processes such as aerobic respiration, aerobic metabolism, and cell respiration. CoQ10 is a nutrient that supports the heart and circulatory system. Although a CoQ10 deficiency is observed in cardiac cases, it is not always the primary cause of cardiopathy; however, it might contribute to the severity of the condition. The efficacy of CoQ10 as a coadjutant in treating heart failure has been studied extensively. CoQ10 supplementation has been shown to enhance ejection fraction due to its role in bioenergetics, its capacity to inhibit plasma LDL oxidation, and its potential to improve endothelial function. Watts *et al*. [11] discovered this effect in individuals with type 2 diabetes [11], while Belardinelli *et al*. investigated it in patients with ischemic heart disease [12]. CoQ10 levels are low in patients with chronic heart failure in blood and myocardial tissue samples [13, 14]. Oral CoQ 10 supplementation improves cardiac contractility and endothelial dysfunction in individuals with stable mild congestive heart failure [15]. The aim of this study was to investigate the impact of Coenzyme Q10 (CoQ10) on trastuzumab-induced cardiotoxicity (TIC) in HER2+3 breast cancer patients.

## MATERIAL AND METHODS

### Study design and participants

The Middle Euphrates Cancer Treatment Institute in Najaf, Iraq, conducted this clinical trial investigation from January 2020 to January 2021. After briefly explaining the treatment strategy, patients provided informed consent to participate. Patients were also told that they could leave the study whenever they wanted. Eligible patients were women diagnosed with breast cancer HER2+ve (HER2+3 or amplified gene) and confirmed by immunohistochemistry (IHC) or fluorescence in situ hybridization (FISH) who had IV trastuzumab injection as the pharmacologic agent in their treatment plan for 1 year. Patients under 18 years, pregnant women, and those with congestive heart failure (CHF), diabetes, statin treatment, stimulants, thyroid dysfunction, or current anticoagulation were excluded from the study. The Superior Committee for Evaluation and Approval of Research Projects of the Faculty of Medicine of the University of Kufa approved all protocols.

### Methods

A total of 100 female breast cancer patients with HER2+ (HER2+3 or amplified gene) were enrolled. Each of these patients met the research requirements. All patients were scheduled to receive traditional adjuvant chemotherapy courses, which comprised 4 cycles of AC (Adriamycin 60 mg/m2, Cyclophosphamide 600 mg/m2), followed by four cycles of T=Taxotere (Docetaxel 75 mg/m2) and H=Herceptin (Trastuzumab 8 mg/kg) loading dose after 3 weeks of rest, then 6 mg/kg maintenance every 3 weeks [16], for 17 cycles. Trastuzumab 440 mg/vial was obtained from Roche in Switzerland.

CoQ10 was administered in oral capsule form at 200 mg from Newton-Everett Nutraceuticals 9160 E.BAHIA DR. SCOTTSDALE, AZ 85260 USA.

The patients were divided into two groups: the control group (50 patients) receiving trastuzumab and placebo for a year, and the CoQ10 treated group (50 patients) receiving Herceptin and 200 mg CoQ10 oral capsules twice daily for a year [17].

### Serum preparation

Blood samples were collected immediately post-treatment, then centrifuged at 1000x g for 15 minutes at 2 to 8°C. The serum fraction was stored at -20°C in the Middle Euphrates Cancer Therapy Center laboratory department in Najaf, Iraq. These serum samples were subsequently employed to assess cTnI, MCP-1, F2-isoprostane, IL-6, and sTLR4 levels using enzyme-linked immunosorbent assays (ELISAs).

### Enzyme immunoassay

Serum levels of cTnI, IL-6, MCP-1, sTLR4, and F2-isoprostane were measured using ELISA kits from Solarbio Co., China.

### Radiographic assessment

The cardiac safety profile of trastuzumab could be improved by addressing early diagnosis of left ventricular (LV) systolic dysfunction via noninvasive cardiac imaging, potentially preventing the negative effects of heart failure [18]. MUGA is a non-invasive nuclear medicine examination used to see the heart's ventricles (lower chambers). It produces a picture of the beating heart on a computer using gamma rays and a radioactive tracer. A MUGA scan is very helpful for assessing the heart's general capacity to pump blood [19]. Nuclear medicine diagnostic procedures like MUGA scans use radioactive isotopes administered to and attached to red blood cells (RBCs). By adding radioisotopes or tracers to RBCs, it is possible to utilize a gamma camera to record the photons produced by these isotopes. In these cameras, a sodium iodide crystal is coupled to photomultipliers, which help convert the energy of the captured photons into an image. Technetium-99m (Tc-99m) is the best radioisotope for MUGA scans since it has a half-life of six hours [20], and the heart receives enough radiation to be detected by the gamma camera. With MUGA scans, many images of the heart were taken at various points in time to create a composite film of numerous cardiac cycles that can be viewed on a computer in two dimensions.

### Statistical analysis

Statistical analysis was conducted using the Statistical Package for the Social Sciences (SPSS) version 20. Descriptive statistics included mean, standard error, and standard deviation. For data analysis, independent-sample t-tests and analysis of variance (ANOVA) were performed. Significance was established at p≤0.05.

## RESULTS

### Effect of Coq10 on MCP1

There was a highly significant decrease (p-value=0.001) in the mean MCP1 levels in the CoQ10-treated group at two stages (3 and 12 months). Additionally, there were statistically significant reductions at baseline, 6 months, and 9 months (p values=0.05, 0.042, and 0.023, respectively) compared to the control group (Table 1).

**Table 1:** Changes in MCP-1 levels in the study and control groups

		Study group		Control group				
MCP1	N	Mean	Std. Error	Mean	Std. Error			
						* T-test	df	p-value
Baseline	50	4.4114	.26288	4.5114	.339	1.609	98	0.05**
After 3 M	50	4.1378	.23966	5.2311	.265	4.794	98	0.001***
After 6 M	50	3.9720	.25372	5.442	.260	1.732	89	0.042**
After 9 M	50	3.5101	.33215	5.897	.305	2.013	89	0.023**
After 12 M	50	3.1936	.24323	5.980	.211	4.388	89	0.001***
Total	250	4.7241	.12353	4.659	.133			

* t-test significant at a0.05.; **Significant; ***Highly significant

### Effect of Coq10 on IL6

Table 2 illustrates the results of the mean IL6 levels between the study and control groups across various data collection stages. Statistically significant decreases in IL6 levels were observed at all stages in the CoQ10-treated group compared to the control group (p<0.05).

**Table 2:** Change in IL6 levels in the treated and control groups

			Study group		Control group				
Biomarkers		N	Mean	Std. Error	Mean	Std. Error	* t-test	df	p-value
IL6	Baseline	50	.061	.010	.062	.000528	42.011	98	0.05*
	After 3 M	50	.056	.001	.064	.000562	469.471	98	0.05*
	After 6 M	50	.048	.001	.067	.000622	499.985	98	0.04*
	After 9 M	50	.042	.001	.069	.012906	29.378	98	0.02*
	After 12 M	50	.040	.001	.071	.007616	51.300	98	0.05*
	Total	250	.053	.002	.056	.003011			

* t-test significant at 0.05.; **Significant

### Effect of Coq10 on sTLR4

As shown in Table 3, the level of sTLR4 mean showed a statistically significant decrease in the CoQ10 treated group compared to the control group across all stages of data collection (p=0.001).

**Table 3:** Change in sTLR4 levels in the treated and control groups

			Study group		Control group				
Biomarkers		N	Mean	Std. Error	Mean	Std. Error	* t-test	df	p-value
TLR4	Baseline	50	6.8342	.160	6.853	.132698	6.832	98	0.001**
	After 3 M	50	6.7294	.133	7.854	.154569	5.739	98	0.001**
	After 6 M	50	6.5558	.142	7.826	.132384	8.840	98	0.001**
	After 9 M	50	6.3216	.134	7.966	.119939	3.491	98	0.001**
	After 12 M	50	6.1498	.179	7.937	.126265	5.592	98	0.001**
	Total	250	6.4481	.067	7.4472	.059980			

* t-test significant at 0.05.; **Highly significant

### Effect of Coq10 on ISO_PGF2α

According to the results of Table 4, there was no statistically significant difference in ISO_PGF2α levels between the treated and control groups at any data collection point (p<0.05).

**Table 4:** Change in ISO_PGF2α levels in the treated and control groups

			Study group		Control group				
	Biomarkers	N	Mean	Std. Error	Mean	Std. Error	* t-test	df	p-value
ISO_PGF2**α**	Baseline	50	743.30	29.839	756.54	31.295	0.156	98	0.438
	After 3 M	50	740.28	30.514	742.28	28.368	0.024	98	0.490
	After 6 M	50	742.54	31.295	746.28	30.514	0.097	98	0.461
	After 9 M	50	747.68	29.575	751.86	29.060	0.101	98	0.460
	After 12 M	50	725.52	30.402	733.30	31.674	0.177	98	0.430
	Total	250	739.86	13.468	746.05	13.407			

* t-test significant at 0.05.

### Effect of CoQ10 on Cardiac Troponin I (cTnI)

The findings presented in Table 5 revealed that CoQ10 contributed to a significant reduction in serum cTnI levels in the treated group compared to the control group. The reduction was significant after 3 months and 9 months (p=0.050, p=0.010, respectively) and highly statistically significant at baseline, 6 months, and 12 months (p=0.018, p=0.008, p=0.008, respectively).

**Table 5:** Change in cTnI levels in the study and control groups

			Study group		Control group				
	Biomarkers	N	Mean	Std. Error	Mean	Std. Error	* t-test	df	p-value
Troponin	Baseline	50	.0266	.00142	.0315	.00184	2.128	98	0.018***
	After 3 M	50	.0281	.00123	.0491	.01186	1.585	98	0.050**
	After 6 M	50	.0292	.00113	.0338	.00150	2.453	98	0.008***
	After 9 M	50	.0294	.00107	.0338	.00150	2.365	98	0.010**
	After 12 M	50	.0294	.00113	.0335	.00121	2.451	98	0.008***
	Total	250	.0285	.00054	.0363	.00246			

* t-test significant at 0.05.; **Significant; ***Highly significant

### Effect of Coq10 on MUGA Scan EF

CoQ10 administration led to a significant decrease in the reduction of EF levels compared to the control group. The decrease was statistically significant after 3 months, 6 months, 9 months, and 12 months (p=0.001, p=0.043, p=0.001, p=0.001, respectively). However, no significant effect on EF levels was observed at baseline (Table 6).

**Table 6:** Change in MUGA EF levels in the study and control groups

			Study group		Control group				
	Biomarkers	N	Mean	Std. Error	Mean	Std. Error	* t-test	df	p-value
Muga. EF	Baseline	50	60.000	.0000	60.020	.020	1.000	98	0.160
	After 3 M	50	59.960	.0400	58.380	.069	5.247	98	0.001***
	After 6 M	50	59.760	.1163	57.660	1.202	1.739	98	0.043**
	After 9 M	50	59.400	.1616	56.720	.230	5.969	98	0.001***
	After 12 M	50	58.660	.3610	55.980	.316	5.585	98	0.001***
	Total	250	59.556	.0880	58.352	.271			

* t-test significant at 0.05.; **Significant; ***Highly significant

## DISCUSSION

Patients who receive trastuzumab treatment for early-stage HER2+ breast cancer have a better prognosis. Cardiotoxicity remains a concern, particularly in the context of curative treatment, and data on its occurrence outside of clinical trials are few. A real-world trial on trastuzumab for cardiotoxicity brought on by HER2-positive breast cancer included more than 3,700 research participants and found a 2.8% incidence of CHF with a 1.0% incidence of severe CHF [21, 22]. In this study, Coenzyme Q10 was utilized to mitigate the cardiotoxicity of trastuzumab. We aimed to explore the possible role of CoQ10 in reducing the cardiac toxicity induced by trastuzumab. Our findings suggested that Coq10 therapy had considerable protective benefits against TIC by mitigating cardiomyocyte damage, decreasing tissue inflammation, and partially restoring normal ejection fraction (EF). Hence, our study underscores the potential therapeutic value of CoQ10 in addressing the challenges of trastuzumab-induced cardiotoxicity.

According to this research, the addition of CoQ10 to the trastuzumab-treated group decreased the cardiotoxic effects of trastuzumab on cardiac muscle. Serial assessments of the MUGA ejection fraction in the control group revealed a considerable decrease. In contrast, patients who received CoQ10 before treatment had a markedly slower decline in ejection fraction. This outcome was in line with other studies [23, 24] that showed that oral CoQ10 treatment significantly increased left ventricular contractility and functional capacity in patients with chronic heart failure compared to placebo. In this study, CoQ10 significantly reduced IL6 levels compared to the control group. This result disagrees with Zhai *et al*., who indicated that CoQ10 had little to no impact on IL6 levels [25], but in accordance with other results [26] indicating that CoQ10 supplementation effectively reduces IL6 levels.

Furthermore, our study showed a significant reduction in MCP-1 levels among the CoQ10-treated group. This effect could potentially be attributed to the inhibitory action of CoQ10 on NFκB, which could reduce the secretion and/or expression of these pro-inflammatory cytokines. This outcome contradicts another study [27], which investigated the effect of CoQ10 on Mcp-1 levels and demonstrated that LPS-induced inflammatory responses were significantly decreased in pre-incubated CoQ10 cells for TNF- α, MIP-1α, and Rantes, respectively, while no significant effect was found for MCP-1. Despite the lack of prior research on the impact of Cq10 on sTLR4 levels, one study [28] investigated the clinical significance of serum sTLR4 in non-small cell lung cancer (NSCLC) and revealed a significant increase in sTLR4 in patients, compared with healthy controls. This study showed a significant decrease in the serial levels of sTLR4 in the treated group compared to the control group. This is also consistent with Cardinale *et al*. [29], who found a relationship between blood sTLR4 levels and tumor stage in NSCLC patients. However, the change in cTNI level did not reach the cutoff level of 0.08 ng/mL (values above this threshold denote elevated cTnI levels) [30]. Our study showed a decrease in serial cTnI levels within the CoQ10-treated group compared to their baseline levels.

Conversely, the control group (without CoQ10 supplementation) had a significant increase in serial cTnI levels compared to baseline. Additionally, we observed a significant reduction in serial cTNI levels within the treated group compared to the control group. This finding is consistent with previous research [30], which reported elevated cardiac troponin I levels in breast cancer patients undergoing trastuzumab monoclonal antibody therapy due to cardiotoxicity. CoQ10 supplementation did not significantly reduce serum F2-isoprostane levels, contrary to previous studies. One potential explanation for this disparity could be attributed to the CoQ10 dosage utilized in our study, 400 mg per day, rather than the 1200 mg recommended by another study [31].

## CONCLUSION

This study found that Coenzyme Q10 provided cardioprotection against trastuzumab cardiotoxicity in patients with Her2+3 breast cancer, as seen by an increase in ejection fraction and a decrease in inflammatory biomarkers and cardiac enzymes.
